# A new evaluation model of a water conveyance channel based on Bayesian theory by integrating monitoring and detection information

**DOI:** 10.1038/s41598-022-12997-6

**Published:** 2022-05-26

**Authors:** Yuan Wang, Zhi-Jian Wei, Jie Ren, Jia-Kun Gong, Di Feng

**Affiliations:** 1 College of Water Conservancy and Hydropower Engineering, Hohai University, Nanjing, 210098 China; 2 College of Mechanics and Materials, Hohai University, Nanjing, 211100 China; 3 College of Civil and Transportation Engineering, Hohai University, Nanjing, 210098 China

**Keywords:** Geomorphology, Solid Earth sciences

## Abstract

Channels are commonly used in long-distance water transfer projects, where landslides, collapses, or erosion may occur in its course of operation; thus, safety evaluation is conducted through monitoring and detection in its key and potentially hazardous areas. However, monitoring and detection information cannot comprehensively reflect the prominent problems of the safety state of the channel in terms of time and space. Therefore, studying how to realize the integration of monitoring and detection information is an important task for the safety evaluations of channels. In this paper, a method of integrating monitoring and detection information based on Bayesian theory is presented. The research shows that the fusion method of gathering monitoring and detection information based on Bayesian theory successfully captures the safety state of high-filling channels, and it can quantify and reduce uncertainty compared with fuzzy theory and the GA-BP neural network. By studying the influence of monitoring information on the safety of the channel, it is found that the horizontal displacement has a greater impact on the safety of the channel than the vertical displacement. A comparison of the results of fusing seven different monitoring points shows that the comprehensive utilization of horizontal and vertical displacement can improve the accuracy of the evaluation results. Compared to the safety coefficient calculated by the actual exploration, the error rate of the GA-BP neural network is 42.7%, and the fusion method based on Bayesian theory is 2.9%. The proposed method based on Bayesian theory can better use the detection information to recognize and understand the rock and soil in advance; hence, the evaluation results are more reliable and consistent with the actual engineering state.

## Introduction

A water diversion project is a powerful means to solve the uneven temporal and spatial distribution of water resources. In many countries, especially in China, the safety evaluation of water diversion projects has been paid great attention by the state. A high-filling channel, which refers to a channel with a filling height of more than 6 m, is a commonly used water conveyance structure in long-distance water transfer projects. For instance, in the Central Line Project of South-to-North Water Diversion, the high-fill channel has a length of about 144 km. In the operation of the high-filling channel, it is affected by the coupling of internal (i.e., material properties) and external (i.e., rainfall and temperature) factors, which may lead to landslide, collapse, and erosion. In order to ensure the safe operation of the high-filling channel, safety monitoring means are usually adopted to grasp its safety status; that is, monitoring facilities such as displacement and stress are installed along the line to obtain its status information, and safety detection (destructive and non-destructive testing) is carried out to explore its internal characteristics and analyze the causes of anomalies.

The safety state of channel engineering is reflected by the safety monitoring and detection over time and space, respectively. The monitoring information of time series can be obtained by safety monitoring, and the slope safety can be evaluated by using the changing characteristics, early warning criterion, or development trend of this information. Some researchers have built safety assessment methods or models by monitoring the internal displacement of the slope^1,2^. It is also believed that monitoring the surface displacement or displacement increment speed of the slope can be used to evaluate its safety^3–7^. Dick et al. judged safety by monitoring the spatial distribution of slope displacement over time in open-pit mines^8^. Monitoring the external environment, such as rainfall, can warn of landslides^9,10^. The above studies have evaluated slope safety from the perspective of the monitoring information changing over time. Engineering detection, mainly including destructive and non-destructive testing, is an essential means to intuitively understand the internal characteristics of a slope, reflect the physical state of the slope in space, and provide a basis for analyzing the causes of abnormal phenomena. Destructive detection determines the distribution of rock and soil by means of excavation and drilling and obtains important information such as the strength parameters of the rock and soil by means of tests. Non-destructive detection techniques, such as acoustic emission^11^, microseismic^12^, high-density electrical^13^, and geological radar^14^ methods, can directly judge the safety state or hidden danger area of a rock slope. To sum up, engineering safety monitoring can obtain continuous time series data, which is convenient for automatic management and safety evaluation. However, it cannot be completely covered due to blind spots in the monitoring. Engineering detection can obtain the engineering performance of specific parts, mainly for engineering defects or abnormal parts, but it has a lack of continuity and cannot cover all areas. Meanwhile, the detection information reflects people’s cognitive level of the rock and soil at a certain time, and the change in the physical quantity (i.e., displacement) of slope monitoring is mainly due to the changes in the rock and soil strength parameters (i.e., cohesion and internal friction angle). Namely, there is a causal relationship between the monitoring and detection information at different times and spaces. Hence, how to realize the fusion of monitoring and detection information is an urgent problem to be solved in project operation management in order to give full play to the respective advantages of monitoring and detection technology in time and space to reflect the physical state and diagnose the hidden danger or health state of the project timely and accurately. There is no research report in the literature on the fusion of monitoring and detection information at home or abroad. Therefore, there is an urgent need to establish a method to make effective use of this valuable information, namely, the fusion of monitoring and detection information to evaluate engineering safety.

Information fusion technology can effectively integrate multiple sources of information and express consistent and accurate results^15^. In order to reduce the loss of original data information, Peng et al. adopted a pixel-level method to fuse monitoring information from multiple sensors to obtain consistent descriptions and interpretations of monitored objects^16^, but it is difficult to integrate different kinds of information. Numerical simulation is a method of calculating the stability of a slope based on a mechanical model, where multi-source information is taken into account in the form of parameters (tolerance weight, cohesion, internal friction angle, etc.)^17^, but it is difficult to consider monitoring information such as displacement. Neural network methods are able to handle complex multi-source information fusion problems with high-dimensional nonlinearity and use their self-learning and reasoning capabilities to learn sample data to achieve predictions^18^. Zhao et al. constructed a BP neural network model to predict landslide hazard levels by combining the monthly rainfall, days of continuous rainfall exceeding 0.1 mm, maximum rainfall within 3 days, water level drop rate, and monitoring information of the reservoir water level^19^. Liu et al. established a dynamic prediction model to predict the slope displacement in the Three Gorges Reservoir area based on the depth learning method^20^, which integrates monitoring information based on time series (i.e., precipitation, reservoir water level, and displacement). Luo et al. constructed a PSO-CA model for predicting slope safety and stability, which utilizes detection information (i.e., cohesion and internal friction angle), weight, slope angle, and height^21^. The application of the neural network method is conditional on having a considerable amount of sample data, and if the sample data are too few, it is difficult for the stability and accuracy of its prediction results to meet the requirements. Meanwhile, it is extremely difficult to obtain a large amount of valuable sample data in reality. Therefore, the fuzzy comprehensive evaluation method is used to solve the problem of multi-source information fusion in slope engineering^22^. The fuzzy comprehensive evaluation method applies the principle of fuzzy transformation to make a general evaluation of objects restricted by various factors^23^. This method avoids the use of sample data and can deal with the fuzzy problems in slope engineering, giving it good suitability. Based on fuzzy theory, engineering geological features and landform features are used to evaluate the slope stability^24,25^, but physical analysis is not involved. The above methods have been well applied and studied in different scenarios of slope engineering. However, in these methods based on physical analysis, the field monitoring (displacement, etc.) and field detection (cohesion and internal friction angle, etc.) information are not easy to fuse to evaluate the safety of high-filling channel slopes. Based on the shortcomings of the above research, this paper carries out relevant research based on Bayesian theory. The innovation of this paper is mainly reflected in the following two aspects. First, based on Bayesian theory, evaluation model for slope safety integrating monitoring and detection information is established. Through the analysis of the uncertainty of the evaluation model and the comparison with numerical simulation, GA-BP neural network and fuzzy comprehensive evaluation methods, the established fusion model has more advantages in dealing with the uncertainty. It can quantify and reduce the uncertainty, its evaluation results are more accurate and the calculation is simpler. Second, this evaluation model is used to study the impact of monitoring point information on channel safety under different time serial numbers. It is found that the horizontal displacement of the channel has a greater impact on channel safety than the vertical displacement.

In this paper, a method of fusing monitoring and detection information based on Bayesian theory is presented. The framework is as follows: (1) construct a Bayesian network by setting the cohesion and internal friction angles as independent variables, and the factors of safety and vertical and horizontal displacements as dependent variables. (2) Calculate the sample data of the response surface function by finite element software; the Bayesian network is quantified by the response surface method. (3) Using Bayesian theory and the Markov chain Monte Carlo algorithm, update the distribution of the independent and dependent variables in the Bayesian network with monitoring and detection information. (4) Use the constructed Bayesian network to realize the fusion of monitoring and detection information of the high-filling channel and evaluate its safety.

## Proposed model of channel safety evaluation by integrating monitoring and detection information based on Bayesian theory

**Figure 1:**
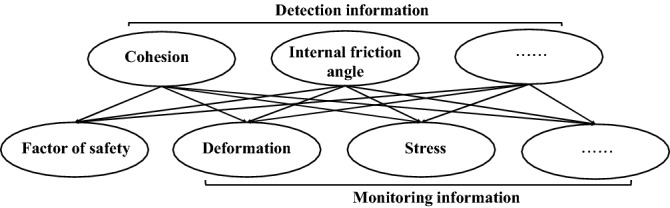
A Bayesian network related to the monitoring information, detection information and factor of safety.

A Bayesian network is a particularly potent model to solve probability problems by logical reasoning and risk analysis. A Bayesian network with independent and dependent variables was constructed based on Bayesian theory^26^ to integrate the monitoring and detection information, as shown in Fig. 1. Independent variables include detection information (i.e., cohesion and internal friction angle). Dependent variables include factors of safety and monitoring information (i.e., displacement).

The detection information is quantified by prior distribution, and the monitoring information is integrated with a likelihood function. Monitoring and detection information is fused in the form of data and expressed as the posterior distribution. Since the posterior distribution is characterized by high-dimensional nonlinearity, it needs to be calculated by the Markov chain Monte Carlo algorithm^27–30^. The algorithm can determine the data (i.e., cohesion and internal friction angle) constrained by the monitoring and detection information. The fusion process of monitoring and detection information can be expressed as follows:

1 $$P(x|d) \, = cL(x|d)P\left( x \right)$$

where *c* is a normalized constant, *P*(*x*) is a priori distribution, which reflects the cognitive level of the soil parameters before obtaining the field monitoring data, *P*(*x*|*d*) is a posteriori distribution, consisting of updated information after the fusion of prior information and field monitoring data, and *L*(*x*|*d*) is a likelihood function.

(1) Likelihood function.

The monitoring value *d* in the likelihood function is linked to the model parameter *x* by a physical model. The numerical model consists of a set of equations and boundary conditions describing the geometry, material properties, and loading conditions^30^. The relationship between the model predictive value *F*(*x*) and the monitoring value *d* is

2 $$d = F\left( x \right) + \varepsilon$$

where *ε* is the deviation between the monitoring value and the predicted value. Without considering the model uncertainty, *ε* is also the observational error of the monitoring value *d*, which is considered to follow the normal distribution.

(2) Markov Chain Monte Carlo (MCMC) simulation.

The Markov chain Monte Carlo (MCMC) simulation method is an effective tool to deal with complex statistical problems^27^. Its core idea is to construct a Markov chain, guide the Markov chain perturbation process according to the transfer rules to approximate the target distribution, and extract the approximate samples to calculate the posterior distribution. MCMC has many algorithms. In this study, we adopted the Metropolis–Hastings (M–H) algorithm, and the main calculation steps are as follows.

1. Generate initial sample θ^0^
2. For *i* = 1 to *n*_*s*_

Generate sample from proposal distribution θ^0^ ~ *g*(θ^*^|*θ*^*i*-1^).

Generate acceptance sample *u* ~ *U*(0,1)

3 $${\text{if}}\,\,\,\,u < Q\left( {{\uptheta }^{i - 1} ,{\uptheta }^{ * } } \right) = \min \left\{ {1,\frac{{f\left( {{\uptheta }^{ * } |y} \right)g\left( {{\uptheta }^{i - 1} |{\uptheta }^{ * } } \right)}}{{f\left( {{\uptheta }^{i - 1} |y} \right)g\left( {{\uptheta }^{ * } |{\uptheta }^{i - 1} } \right)}}} \right\}$$

θ^*i*^ = θ^*^

otherwise,

θ^*i*^ = θ^*i*-1^.

θ_0_ is an initial vector value of the model parameters to be estimated, *n*_*s*_ is the total number of samples, and *f* (θ| *y*) is the target distribution that is the posterior distribution after the Bayesian update. *g*(θ^*^| θ^*i*-1^) is the selected proposal distribution used when a new sample θ^*^ is conditional on the previous point θ^*i*-1^. According to the Metropolis algorithm, *g*(θ^*i*-1^| θ^*^) must be chosen as symmetrical to keep the calculations efficient, so *g*(θ^*i*-1^| θ^*^) is the same as *g*(θ^*^| θ^*i*-1^). *Q*(θ^*i*-1^,θ^*^) can be simplified to

4 $$Q\left( {{\uptheta }^{i - 1} ,\theta^{ * } } \right) = \min \left\{ {1,\frac{{f\left( {{\uptheta }^{ * } |y} \right)}}{{f\left( {{\uptheta }^{i - 1} |y} \right)}}} \right\}$$

The above calculation process is further analyzed. If the value of *Q*(θ^*i*−1^,θ^*^) is greater than a randomly generated sample *u* from *U*(0,1), θ^*^ is accepted as a new sample. There are two possibilities. First, when the ratio of *f*(θ^*^| *y*) to *f*(θ^*i*-1^| *y*) is greater than 1, the new sample θ^*^ is accepted since *Q*(θ^*i*-1^,θ^*^) is always 1. Second, when the ratio of *f*(θ^*^| *y*) to *f*(θ^*i*-1^|*y*) is less than 1, the acceptance of the new sample θ^*^ depends on the random sample *u* and the ratio of *f*(θ^*^| *y*) to *f*(θ^*i*-1^| *y*). If the new sample h is not accepted as an *i*th sample, the (*i*-1)th sample becomes the *i*th sample. After sufficient iterations, this produces samples that approximate the target distribution.

**Figure 2:**
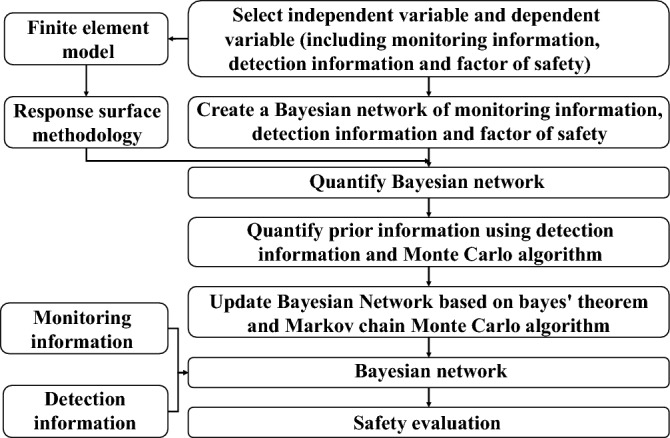
Bayesian network framework for fusion of monitoring and detection information.

The main steps of integrating monitoring and detection information based on Bayesian theory are as follows, and the framework is shown in Fig. 2.

1. A Bayesian network is constructed to correlate the monitoring information, detection information, and factors of safety, as shown in Fig. 1.
2. The monitoring information is obtained manually or by sensors, the detection information is obtained by excavation or drilling and combined with laboratory tests, and the prior distribution of detection information is obtained by statistical analysis.
3. The response surface function^31^ quantifies the relationship between the Bayesian network models. The sample data of the response surface function are calculated by the finite element software, and the response surface coefficient is obtained by fitting the sample data.
4. The prior distribution of the monitoring information is obtained by applying the Monte Carlo algorithm to the response surface function.
5. Based on Bayesian theory^30,32^ and the Markov chain Monte Carlo algorithm (Metropolis–Hastings)^27,33–35^, a program is developed to run the Bayesian network models.
6. The Bayesian network is updated with the monitoring or detection information to obtain the distribution of cohesion and internal friction angle, and then the distribution of the updated factors of safety is calculated.

## Example analysis

**Figure 3:**
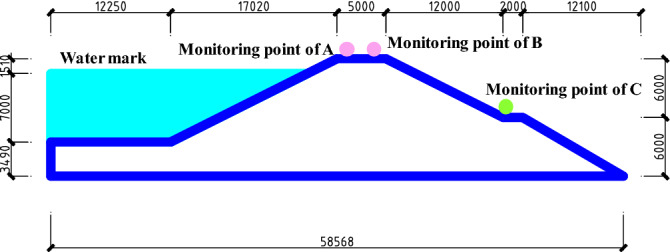
Monitoring section H of high filling channel. Note: The units in the picture are millimeters.

Through field investigation and data sorting, a section with cracks at the top of the high-filling channel was selected as a typical section, and half of the section was selected as the research object, named the H section, as shown in Fig. 3. The high-filling channel has a height of 12 m, and the crest of the channel has a width of 5 m. The inner and outer slope ratio of the crest is 1:2. A grade I berm with a width of 2 m was set 6 m from the crest of the channel. The water level in the high-fill channel was 7 m high, and the load on the crest was 20 kpa. The cohesion (*c*) and internal friction angle (*φ*) of the high-filling channel were set as two random variables.

The high-filling channel slope was calculated and analyzed according to the homogeneous rock and soil mass, and the main parameters of rock and soil mechanics are shown in Table 1. Three monitoring points were set up to capture the safety state of the slope: the vertical displacement at point A(*Y*_A_), the vertical displacement at point B (*Y*_B_), and the horizontal displacement at point C (*X*_C_), as shown in Fig. 3.

**Table 1:** Main parameters of rock and soil mechanics.

Main parameters of rock and soil	Value
Cohesion (kPa)	N (24.4, 5)
Internal friction angle (°)	N (18.5, 4)
Young’s modulus (MPa)	20
Unit weight (kN/m^3^)	19.6
Poison’s ratio	0.3

N(*μ*, *σ*) indicates that the mean value of normal distribution is *μ* and the standard deviation is *σ*. For the values of cohesion and internal friction angle, see “Safety evaluation of high-filling channel slope with monitoring and detection information” of this article.

### Constructing a Bayesian network

**Figure 4:**
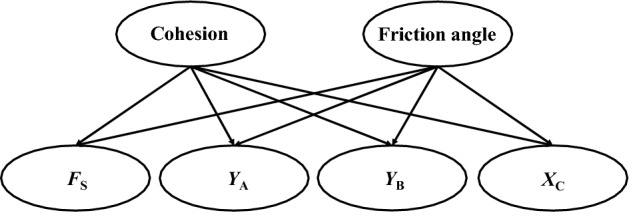
A Bayesian network related to the monitoring information, detection information and factor of safety. *F*_S_ = factor of safety, *Y*_A_ = vertical displacement at point A, *Y*_B_ = vertical displacement at point B, *X*_C_ = horizontal displacement at point C.

Cohesion and the internal friction angle are the main indicators to evaluate slope safety, so in this study, we selected the cohesion (*c*) and internal friction angle (*φ*) in the detection information as independent variables, and the factors of safety and monitoring information as dependent variables to construct a Bayesian network that had six nodes and eight directed arcs. The cohesion and internal friction angle are the parents of the Bayesian network, while the factors of safety, *Y*_A_, *Y*_B_, and *X*_C_, are the children of the Bayesian network. A change in any of the variables will result in changes in the node distribution (probability distribution) in the whole network. The constructed Bayesian network is shown in Fig. 4.

The factors of safety and monitoring and detection information of the high-filling channel slope were the performance indicators of the mechanic parameters of the materials. A change in the mechanical parameters of the rock and soil materials will lead to changes in the factors of safety and monitoring information. According to this principle, an explicit polynomial was constructed to quantify the relationship between the nodes^31^.

### Quantification of the Bayesian network

The quantification of the Bayesian network requires an a priori probability distribution of the basic nodes (without parents) and other nodes (with parents) in Fig. 4. The basic nodes refer to the cohesion (*c*) and internal friction angle (*φ*), and the other nodes refer to the factors of safety and displacement (*Y*_A_, *Y*_B_, and *X*_C_). The mathematical distribution of the basic node is given by the field geological exploration data and statistical analysis. In this section, finite element analysis software and response surface method are used to quantify the prior distributions of *F*_S_, *Y*_A_, *Y*_B_, and *X*_C_.

**Figure 5:**
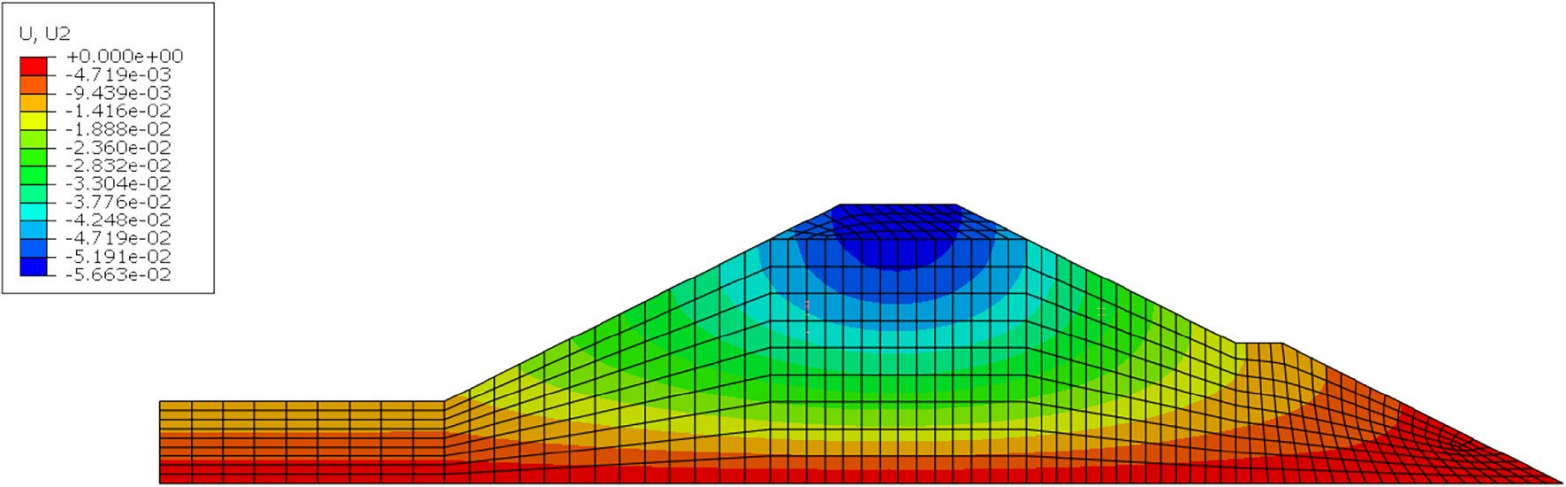
Nephogram of vertical displacement of high filling channel.

A finite element model of the section in Fig. 3 was constructed by using the finite element software to calculate the displacement of a typical section of the high-filling channel slope under different cohesion and internal friction angle values. The safety factor was calculated based on the strength reduction method. The slope was in a state of plane strain, so a two-dimensional finite element model was established for the calculation and analysis. In this model, the horizontal displacement was constrained by the left boundary, and the horizontal and vertical displacement was constrained by the lower boundary. The eight-node quadrilateral element was used to divide the mesh, and the calculation model adopted the Mohr–Coulomb criterion. In this section, 25 combinations of *c* and *φ* needed to be calculated, that is, the combination of *u*, *u* ± *σ*, and *u* ± 2*σ*, where *u* and *σ* are the mean and standard deviation of *c* and *φ*, respectively. The values of the model parameters are shown in Table 1. Figure 5 shows that the vertical displacement nephogram of the high-filling channel was calculated by using the finite element software when the cohesion was 24.4 kPa and the internal friction angle was 18.5°.

The calculated data were used to fit the functions of the factors of safety (*F*_S_) and displacements (*Y*_A_, *Y*_B_, *X*_C_) on the cohesion (*c*) and the internal friction angle (*φ*), respectively. In this paper, the cubic polynomials of the cohesion (*c*) and internal friction angle (*φ*) are used to express the factors of safety and displacements (*Y*_A_, *Y*_B_, *X*_C_), as shown in Formula (5).

5 $$f\left( {x,y} \right) \, = p_{00} + p_{{{1}0}} x + p_{{0{1}}} y + p_{{{2}0}} x^{{2}} + p_{{{11}}} xy + p_{{0{2}}} y^{{2}} + p_{{{3}0}} x^{{3}} + p_{{{21}}} x^{{2}} y + p_{{{12}}} xy^{{2}} + p_{{0{3}}} y^{{3}}$$

where *f*(*x,y*) is the objective function (factor of safety or displacement), *p*_00_, *p*_10_, and so on, are the undetermined coefficients, and *x,y* is the value of the cohesion (*c*) and internal friction angle (*φ*). The response surface coefficients and the determination coefficient *R*^2^ are shown in Table 2.

**Table 2:** Coefficients for response surface.

Coefficient	*F*_*S*_	*Y*_*A*_	*Y*_*B*_	*X*_*C*_
*p*_00_	0.1841	− 0.3147	− 0.3637	0.9055 × 10^–1^
*p*_10_	0.1745 × 10^–1^	0.1323 × 10^–1^	0.1628 × 10^–1^	− 0.4178 × 10^–2^
*p*_01_	0.3733 × 10^–1^	0.2069 × 10^–1^	0.2395 × 10^–1^	− 0.6718 × 10^–2^
*p*_20_	0.7999 × 10^–3^	− 0.2325 × 10^–3^	− 0.2976 × 10^–3^	0.6474 × 10^–4^
*p*_11_	0.3712 × 10^–3^	− 0.6579 × 10^–3^	− 07,833 × 10^–3^	0.2217 × 10^–3^
*p*_02_	0.1949 × 10^–3^	− 0.5563 × 10^–3^	− 0.6296 × 10^–3^	0.1714 × 10^–3^
*p*_30_	− 0.9871 × 10^–5^	0.1440 × 10^–5^	0.1922 × 10^–5^	− 0.2995 × 10^–6^
*p*_21_	− 0.1268 × 10^–4^	0.5295 × 10^–5^	0.6534 × 10^–5^	− 0.1764 × 10^–5^
*p*_12_	0.1217 × 10^–4^	0.8603 × 10^–5^	0.9957 × 10^–5^	− 0.2883 × 10^–5^
*p*_03_	− 0.3362 × 10^–5^	0.4961 × 10^–5^	0.5505 × 10^–5^	− 0.1392 × 10^–5^
*R*^2^	0.9995	0.9886	0.9863	0.9377

### Safety evaluation of high-filling channel slope with monitoring and detection information

**Step 1:** Acquisition of prior information of rock and soil strength parameters.

In this study, it was considered that the cohesion and internal friction angle obeyed normal distribution, the relevant parameters of which were obtained from a field investigation report and statistical analysis. The geological exploration report shows that the low standard strength parameter of the consolidated undrained shear was *c* = 40.8 kPa, *φ* = 20.2. Due to the crack in the channel embankment, the maximum width of the crack on the crest reached 2 cm, the filling of the canal embankment was mixed with expansive soil, and hence, the filling material was uneven and the compactness of part of the channel embankment filling body was slightly insufficient. Therefore, it was necessary to modify the value of the soil strength parameter of the channel embankment, and the value of the strength parameter of the embankment body was reduced on the basis of the low standard of the experimental value. According to the engineering data, the reduction coefficients of the expansive soil strength parameters *c* and *φ* are *α* and *β*, respectively, where *α* is 0.4 ~ 0.8 and *β * is 0.9 ~ 0.95. Taking *α* = 0.6 and *β* = 0.92 into comprehensive consideration, after reduction, the strength parameter *c* (reduction) was 24.4 kPa and *φ* (reduction) was 18.5°, that is, the mean value of cohesion of the high-filling channel slope was 24.4 kPa, and the mean value of the internal friction angle was 18.5°. Through statistical analysis, it was concluded that the standard deviation of cohesion was 5 kPa, and the standard deviation of internal friction angle was 4°.

**Step 2:** Quantification of prior information using Monte Carlo.

**Figure 6:**
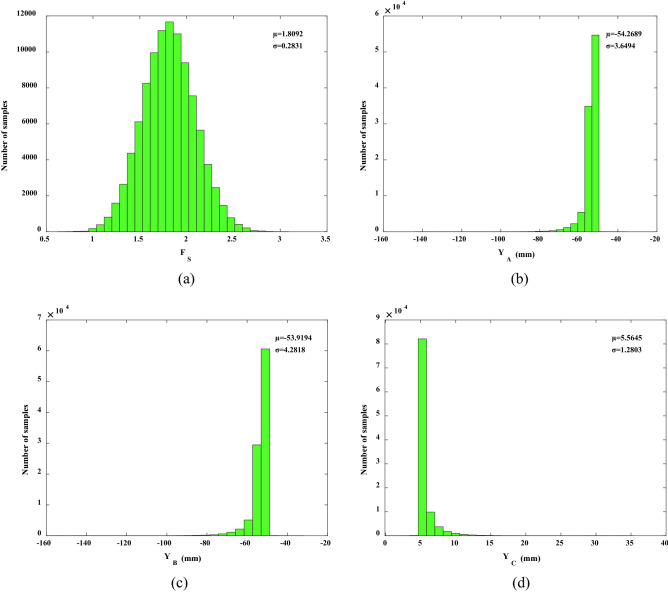
Prior distributions of: (**a**) *F*_S_; (**b**) *Y*_A_; (**c**) *Y*_B_ and (**d**) *X*_C_.

Using Formula (2), constructed with the response surface method, the initial distribution of the cohesion and internal friction angle was simulated by Monte Carlo 100,000 times to obtain the prior distributions of *F*_S_, *Y*_A_, *Y*_B_, and *X*_C_, as shown in Fig. 6a–d. The mean and standard deviation of *F*_S_ were 1.8092 and 0.2831; the mean and standard deviation of *Y*_A_ were − 54.2689 mm and 3.6494 mm; the mean and standard deviation of *Y*_B_ were − 53.9194 mm and 4.2818 mm; the mean and standard deviation of *X*_C_ were 5.5645 mm and 1.2803 mm. The *F*_S_ in Fig. 6a only shows the basic distribution without any monitoring information. With the monitoring information of *Y*_A,_ *Y*_B_, and *X*_C_, the *F*_S_ can be updated using the Bayesian network shown in Fig. 4. In this study, it was assumed that the model errors of *Y*_A_, *Y*_B_, and *Y*_C_ followed a normal distribution N(0, 0.25*u*), where *μ* represents the estimated mean of the predicted value *F*(*x*)^26^. These were used in the likelihood function.

**Step 3:** Integration of field monitoring information.

As shown in Figs. 2 and 4, the Bayesian network uses the response surface function to quantify the relationship between the network nodes. After obtaining the field monitoring information, the network node distribution is updated based on Bayesian theory and the Markov chain Monte Carlo algorithm. In the MCMC calculation process, the sampling weights of the cohesion and internal friction angle were 7 and 6, respectively, and there were 120,000 sampling times. When using the sampling data to calculate the cohesion and internal friction angle, the data after the sampling that were stable were selected for calculation, and the first 25,000 samples did not participate in the calculation^35^. The following is the fusion of the monitoring information obtained on site. Based on the monitoring information from January 13, 2018 (*Y*_A_ = 39.5 mm, *Y*_B_ = 31.4 mm, *X*_C_ = 11.29 mm), the mean value of cohesion was 19.5571 kPa, the standard deviation was 4.3278 kPa, the mean value of internal friction angle was 13.6398°, the standard deviation was 3.1174°, the mean value of the factor of safety was 1.3755, and the standard deviation was 0.2228, calculated and updated by the Bayesian network. After the monitoring information is obtained by Bayesian network, the strength parameters of rock and soil are obtained by back analysis, and the distribution of factors of safety can be updated based on the results of the back analysis.

## Results and discussion

### Features of this method

Compared to previous studies^17,19–21,36^, the proposed method of using monitoring and detection information for slope safety evaluation of high-filling channels has the following characteristics:

1. Monitoring and detection information can be fused into a single model. For fusing monitoring and detection information, the Bayesian method is a very powerful tool. Different from other methods, the method can consider the field detection information in the form of prior information, and then fuse the monitoring information by way of parameter inversion based on numerical simulation analysis. Monitoring and detection information can update the network and obtain security assessments.
2. Model uncertainty can be considered. The method proposed in this paper uses MCMC simulations to deal with the uncertainties associated with Bayesian networks. Under the constraints of monitoring and detection information, a large number of samples are sampled by the MCMC method so that the theoretical solution is maximally close to the real solution to reduce the uncertainty, and the evaluation results will be more reliable.

### Accuracy verification of the fusion method

The typical section H was analyzed, and the change in the safety state of the high-filling channel slope before reinforcement was studied. Based on the monitoring data from January 13, 2018 to May 5, 2018, the Bayesian network was used to update and comparatively analyze the distribution of the cohesion, internal friction angle, and factor of safety of the high-filling channel slope. The field monitoring data are shown in Table 3.

**Table 3:** Field monitoring of displacement at points A, B and C.

Time (year/month/day)	Monitoring time sequence number	*Y*_A_ (mm)	*Y*_B_ (mm)	*X*_C_ (mm)
2018/1/13	1	39.5	31.4	11.29
2018/2/1	2	41.1	30.5	10.49
2018/3/17	3	43.2	33.6	7.69
2018/3/24	4	43.2	33.6	10.19
2018/3/31	5	43.2	33.6	11.66
2018/4/7	6	45.3	36.2	11.29
2018/4/14	7	45.3	36.2	13.09
2018/4/21	8	45.3	36.2	13.99
2018/4/28	9	45.3	36.2	14.29
2018/5/5	10	46.4	37.6	15.59

**Figure 7:**
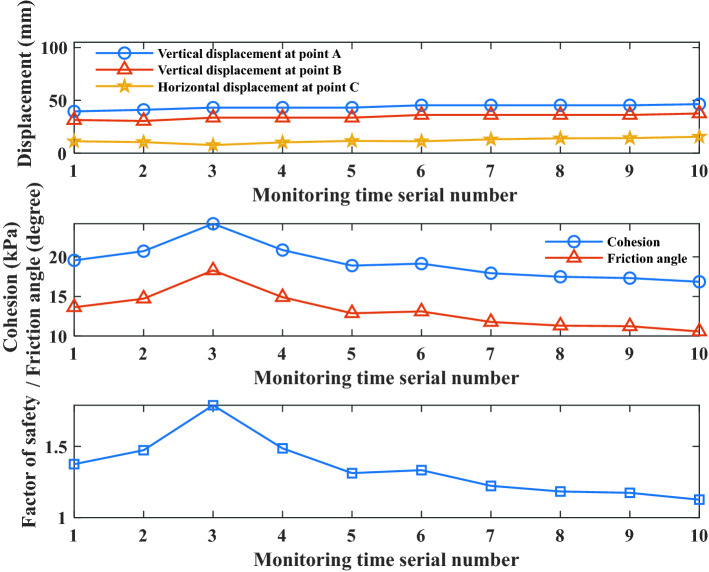
The mean values of cohesion, internal friction angle and factor of safety obtained by updating the field monitoring information of displacements at point A, B and C under different time serial numbers.

As shown in Table 3 and Fig. 7, changes in the displacements of points A, B, and C (Table 3) led to changes in the cohesion, internal friction angle, and factor of safety. From the time sequence numbers 2 to 3, the vertical displacement at point A increased by 2.1 mm, the vertical displacement at point B increased by 3.1 mm, the horizontal displacement of point C decreased by 2.8 mm, and both the cohesion and internal friction angle increased. It can be seen that the cohesion and internal friction angle were affected by the multi-point displacement. The safety factor also increased with the cohesion and internal friction angles, with the average value increasing from 1.4735 to 1.7897. This shows that the safety factor was jointly affected by the displacements of multiple points. Further analysis of the change in displacement shows that the increase in the vertical displacements of points A and B was greater than the decrease in the horizontal displacements. Normally, safety factors should have been reduced, but they increased, indicating that horizontal displacement had a greater impact on the safety factors. From the time sequence numbers 3–5, the factor of safety decreased gradually; from the time sequence numbers 5–6, the factor of safety increased; after the time sequence number 6, the factor of safety decreased gradually. This shows that the safety factor also changed with the change in external factors at different times of the channel. The safety factors (mean values) of time numbers 8, 9, and 10 were 1.1834, 1.1740, and 1.1261, respectively. According to the fact that the slope was safe if *F*_S_ was larger than 1.2, the slope was less safe if 1.0 ≤ *F*_S_ ≤ 1.2, and the slope was unsafe if *F*_S_ < 1^37,38^. The factors of safety (mean values) of the time numbers 8, 9, and 10 were 1.1834, 1.1740, and 1.1261 respectively, indicating that the slope of the high-filling channel was in a less safe state. This result was confirmed by the increased length of the cracks that appeared at the top of the channel. Therefore, it is necessary to pay close attention to reinforcing the high-filling channel slope so as to prevent it from failures due to insufficient safety reserves.

### Uncertainty analysis of interval distribution of variables

**Figure 8:**
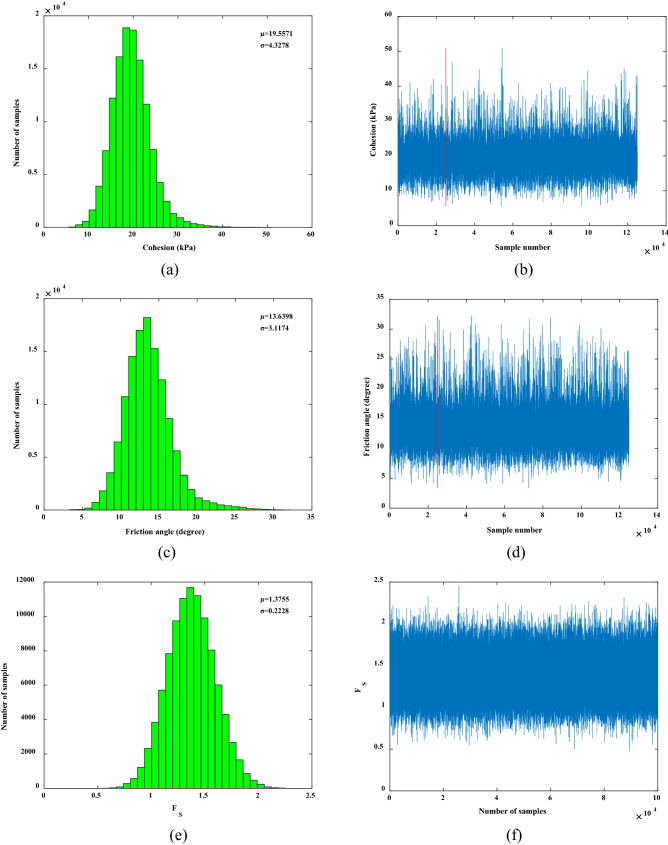
Samples for MCMC simulation: (**b**) *c*; (**d**) *φ* and (**f**) *F*_S_; and the corresponding histograms (**a**) *c*, (**c**) *φ* and (**e**) *F*_S_. The first 25% of the samples in (**b**) and (**d**) are not the final calculation and analysis samples, and the actual sampling times are 1.25 times of the planned sampling times.

The monitoring information from January 13, 2018 and May 5, 2018 in Table 3 was selected for research and analysis. The monitoring information of displacement at the monitoring point on January 13, 2018 was put into the Bayesian network. The mean value of cohesion *c* was 19.5571 kPa, and the standard deviation was 4.3278 kPa; the mean value of the internal friction angle *φ* was 13.6398°, and the standard deviation was 3.1174°. By inputting the updated cohesion and internal friction angle into the Bayesian network, the mean value of the factor of safety of the high-filling channel slope was 1.3755 and the standard deviation was 0.2228. Figure 8a,c,e shows the distributions of the cohesion, internal friction angle, and factor of safety, respectively. The sampling trajectories of the cohesion, internal friction angle, and factor of safety in Fig. 8b,d,f show that the sample of the MCMC moved randomly and reached a steady state, and the sampling trajectory of the factor of safety was the most stable, which was verified by the standard deviation of the three as well.

**Figure 9:**
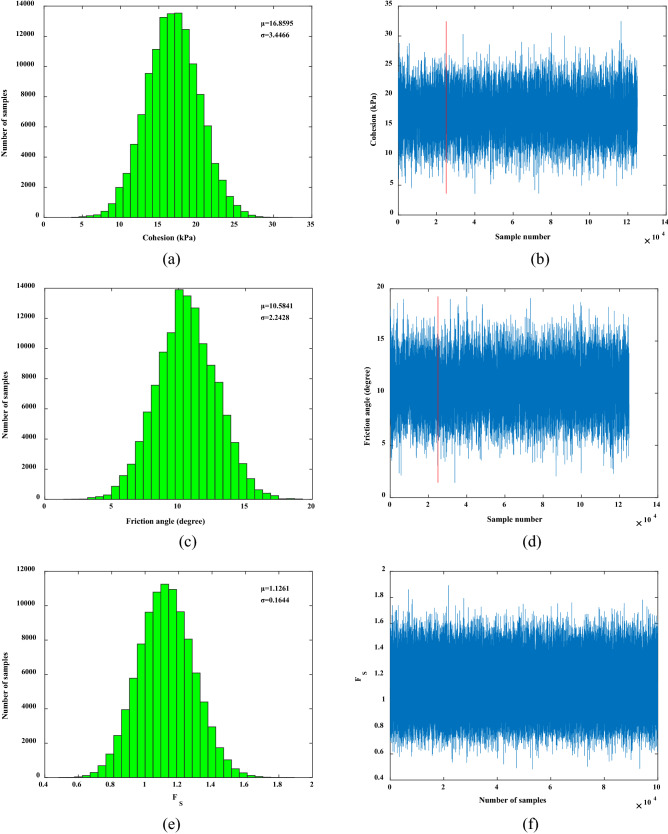
Samples for MCMC simulation: (**b**) *c*; (**d**) *φ* and (**f**) *F*_S_; and the corresponding histograms (**a**) *c*, (**c**) *φ* and (**e**) *F*_S._ The first 25% of the samples in (**b**) and (**d**) are not the final calculation and analysis samples, and the actual sampling times are 1.25 times of the planned sampling times.

The monitoring information of displacement at the monitoring point on May 5, 2018 was input into the Bayesian network. It was calculated that the mean value of cohesion *c* was 16.8595 kPa, the standard deviation was 3.4466 kPa, the mean value of the internal friction angle *φ* was 10.5841°, and the standard deviation was 2.2428°. Then, the mean value of the factor of safety of the high-filling channel slope was 1.1261, and the standard deviation was 0.1644. Figure 9a,c,e shows the distributions of the cohesion, internal friction angle, and factor of safety, respectively. Figure 9b,d,f shows the sampling trajectories of the cohesion, internal friction angle, and factor of safety, respectively.

**Figure 10:**
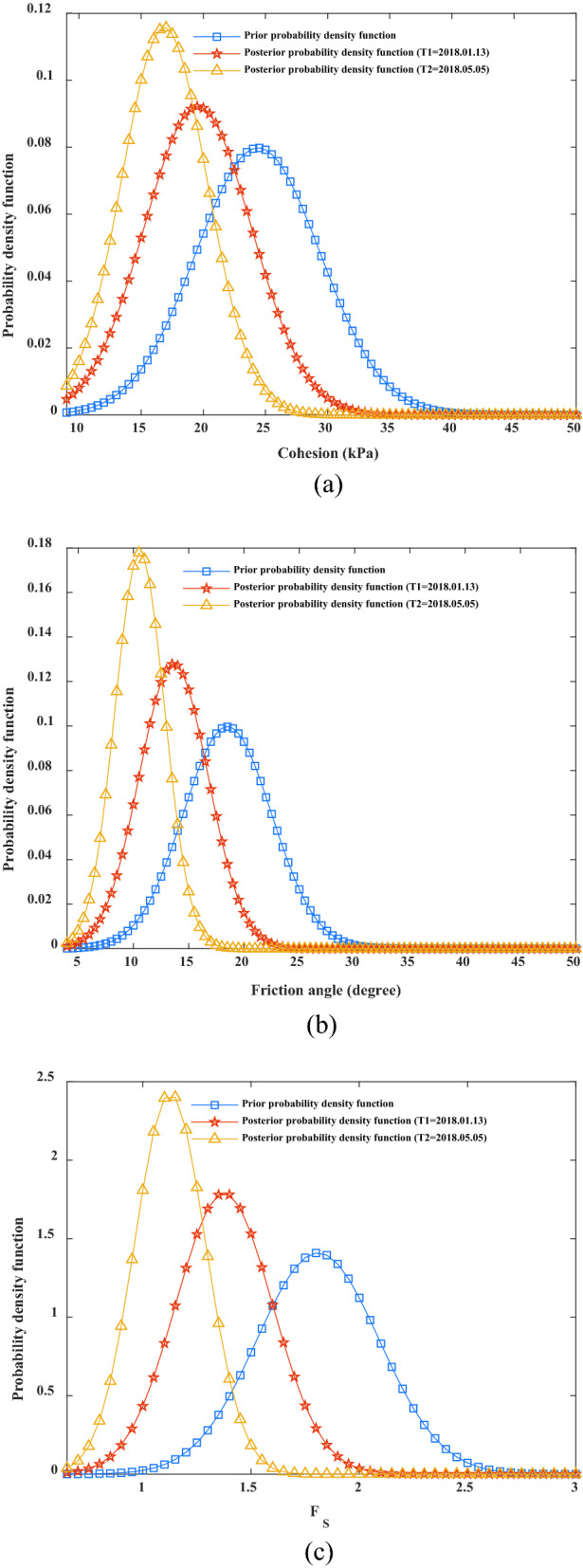
A posteriori probability density function of *c*, *φ* and *F*_S_ after obtaining monitoring information.

Based on the calculated results from January 13 and May 5, 2018, Fig. 10a–c shows the distributions of the posterior probability density functions of the cohesion, internal friction angle, and factor of safety, respectively. The distributions of the prior probability density function of the cohesion, internal friction angle, and factor of safety are also included in the diagram for comparison. As shown in Fig. 10a–c, compared to the distribution of a prior probability density function, the distribution of a posterior probability density function of the cohesion, internal friction angle, and factor of safety was more concentrated, the discreteness was smaller, and the mean value and standard deviation were obviously reduced, indicating that after the monitoring information was obtained, the distributions of the cohesion, internal friction angle, and factor of safety had been updated. When the monitoring displacements (*Y*_A_ = 39.5 mm, *Y*_B_ = 31.4 mm, and *X*_C_ = 11.29 mm) were obtained, the mean value and variance of cohesion decreased from 24.4 kPa and 5 kPa to 19.5571 kPa and 4.3278 kPa, respectively, and the mean value and standard deviation of the internal friction angle decreased from 18.5° and 4° to 13.6398° and 3.1174°, respectively. Thus, it can be seen that the uncertainty of rock and soil parameters near the monitoring point can be reduced by integrating monitoring information^39^. The mean value and variance of the factor of safety decreased from 1.8092 and 0.2831 to 1.3755 and 0.2228, respectively. When the monitoring displacements (*Y*_A_ = 46.4 mm, *Y*_B_ = 37.6 mm, and *X*_C_ = 15.59 mm) were obtained, the mean value and variance of cohesion decreased from 24.4 kPa and 5 kPa to 16.8595 kPa and 3.4466 kPa, respectively, and the mean value and standard deviation of the internal friction angle decreased from 18.5°and 4° to 10.5841°and 2.2428°, respectively. The mean value and variance of the factor of safety decreased from 1.8092 and 0.2831 to 1.1261 and 0.1644, respectively. With the increases in *Y*_A,_ *Y*_B_, and *X*_C_, the factor of safety decreased, which is in accordance with the basic law of deformation and safety stability of the channel slope, which is also similar to the study of Peng et al.^26^. If both the cohesion and internal friction angle obey other distributions (i.e., logarithmic normal distribution), the fusion method constructed in this study can also be used to infer the posterior distribution of the cohesion and internal friction angle and update the factor of safety based on the monitoring and detection information.

### Influence analysis of information from different monitoring points on the factor of safety based on the fusion method

We selected the monitoring information of the high-filling channel slope in Table 3, namely, *Y*_A_ = 46.4 mm, *Y*_B_ = 37.6 mm, and *X*_C_ = 15.59 mm. Based on the constructed Bayesian network, in this study, we explored the influence of the displacements of different monitoring points on the factor of safety and designed the following simulation test scheme. Namely, we integrated the displacements of only point A, point B, or point C, points A and B, points A and C, points B and C, and points A, B, and C on the factors of safety of the high-filling channel slope. The calculated results are shown in Table 4.

**Table 4:** Mean value and standard deviation of updated cohesion, internal friction angle and factor of safety after obtaining monitoring information.

Monitoring displacement (mm)		Updated cohesion and internal friction angle				Updated factor of safety	
		Cohesion (MPa)		Friction angle (degree)			
		Mean value (*μ*)	Standard deviation (*σ*)	Mean value(*μ*)	Standard deviation (*σ*)	Mean value (*μ*)	Standard deviation (*σ*)
*Y*_A_ = 46.4	Vertical displacement	24.6961	4.8580	18.8590	3.7978	1.8386	0.2736
*Y*_B_ = 37.6	Vertical displacement	24.7904	4.7356	18.9480	3.7128	1.8465	0.2682
*X*_C_ = 15.59	Horizontal displacement	15.7990	3.8790	9.6077	2.5022	1.0428	0.1812
*Y*_A_ = 46.4 *Y*_B_ = 37.6	Vertical displacement	24.9837	4.6839	19.0552	3.5758	1.8613	0.2421
*Y*_A_ = 46.4 *X*_C_ = 15.59	Vertical and horizontal displacement	15.9540	3.7403	10.1894	2.3996	1.0749	0.1752
*Y*_B_ = 37.6 *X*_C_ = 15.59	Vertical and horizontal displacement	16.7107	3.5588	10.1205	2.3249	1.0988	0.1692
*Y*_A_ = 46.4 *Y*_B_ = 37.6 *X*_C_ = 15.59	Vertical and horizontal displacement	16.8595	3.4466	10.5841	2.2428	1.1261	0.1644

As shown in Table 4, the updated factors of safety, calculated by combining the information of different monitoring points, are different. Only by fusing the monitoring information of point A, point B, or point C respectively, the mean values of the factors of safety were 1.8386, 1.8465, and 1.0428. The standard deviations were 0.2736, 0.2682, and 0.1812 respectively. According to the previous results—if *F*_S_ > 1.2, the slope is safe, if 1.0 ≤ *F*_S_ ≤ 1.2, the slope is less safe, and if *F*_S_ < 1, the slope is unsafe—the high-filling channel slope was in the safe state only by fusing the monitoring information of point A or B, respectively. However, when fusing the monitoring information of point C, the mean value of the factor of safety was 1.0428, and hence, it can be judged that the high-filling channel slope was about to be unsafe, which is not consistent with the safety state of the field high-filling channel slope. Therefore, there was great uncertainty in the safety state of the high-filling channel slope if the monitoring information of only point A, B, or C was integrated. Combining the monitoring information of point A and point B, the mean value of the factor of safety was 1.8613, indicating that the high-filling channel slope was in a safe state; however, this is not consistent with the cracks at the top of it. When point C was fused with the displacements of point A and point B, respectively, the mean values of the factor of safety were 1.0749 and 1.0988, indicating that the high-filling channel slope was less safe, which is consistent with the phenomenon of the cracks at the top of it. By combining the monitoring displacements of points A, B, and C altogether, the mean value of the factor of safety was 1.1261, indicating that the high-filling channel slope was less safe; this result is also consistent with the phenomenon of the cracks at the top of it. From the field operation status, it can be seen that the monitoring information of the fusion of points A, B, and C is more in line with the actual state. The above results show that the factor of safety calculated by fusing the vertical and horizontal displacements is consistent with the actual abnormal phenomenon on site, and the result calculated by the fusion of only the vertical or horizontal displacement is not consistent with the actual abnormal phenomenon. These results show that engineers need to pay attention to both the vertical and horizontal displacements. The more the monitoring information of the vertical and horizontal displacements is fused, the closer the fusion results are to the real state. The prediction of the safety of the high-filling channel slope will be inaccurate by only relying on the monitoring information of the vertical or horizontal displacement. Yang et al. also stated that with less monitoring information, the fusion effect will become worse^40^.

According to the actual operation state of the site, no instability failure occurred during the reinforcement of the high-filling channel slope, indicating that the high-filling channel slope is not in a critical failure state. The cracks at the top of the high-filling channel slope are caused by insufficient safety reserves, which also verifies the correctness of the Bayesian network fusion results. A slope is a system of engineering, and it is one-sided to use only vertical or horizontal displacement to evaluate its safety state. The Bayesian method can effectively integrate various information^26^. Therefore, the Bayesian network can be used to integrate monitoring information to realize the safety evaluation of high-filling channel slopes.

### Comparison with the present research

#### Comparison with fuzzy theory approaches

The research of Huang et al. showed that the corresponding membership function can be established for multi-source information based on fuzzy theory to obtain the membership degrees of all the information, and then the criteria of fuzzy theory can be used to fuse the information to realize the safety evaluation of the slope^24^. According to the principle of the maximum membership degree, the method selects the safety state corresponding to the maximum probability as the evaluation result. The comprehensive evaluation membership vector calculated in^24^ is (0.2061, 0.5504, 0.2270, 0.0165), and the safety state corresponding to the maximum probability (0.5504) is the evaluation result. Due to the use of probability to characterize the results, the evaluation results have uncertainty. The method used in this study was to take the uncertainty into account in the sample sampling. When the displacement information is fused, the uncertainty of the evaluation results is reduced. Secondly, the method proposed in this paper is a fusion method based on the mechanical model to realize the fusion of monitoring and detection information, which has a clear physical meaning. The model does not use membership functions to approximate the monitoring and detection information. There is also no human subjective factor involved in the safety evaluation, which avoids the influence of this factor on the evaluation results. Finally, the method used in the study in^24^ was to fuse multi-source information at a certain time, while the method proposed in this paper is to realize the integration of monitoring and detection information under the premise of the cognition of historical information, which makes full use of the important values of historical information.

#### Comparison of evaluation results between the fusion method and the BP neural network

(1) Comparative analysis of algorithm complexity.

The complexity of the algorithm mainly includes complexity of time and space. In terms of time complexity, under the same computer configuration, GA-BP neural network completes 2500 training times, and the calculation time required for training is 60 s. The fusion method based on Bayesian theory takes 100 s to calculate 100,000 iterations, which is more accurate than neural network. In terms of spatial complexity, GA-BP neural network occupies more storage space, including the space occupied by the algorithm itself and the additional auxiliary space. The auxiliary space used at runtime is the key factor to measure the complexity of space. It can be seen that the complexity of Bayesian fusion algorithm is less than that of GA-BP neural network.

(2) Comparative analysis of calculation results.

**Figure 11:**
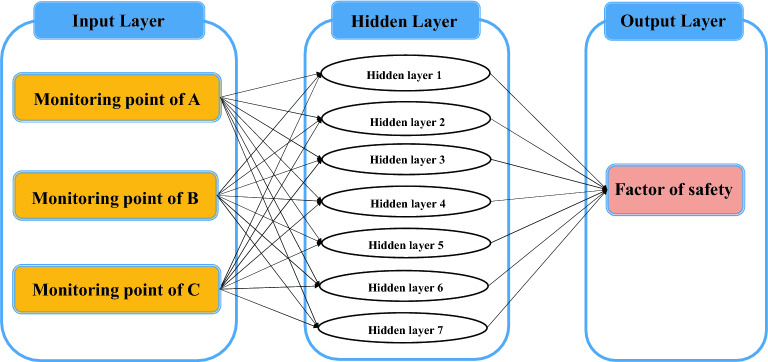
Safety evaluation and prediction model of high filling channel slope.

The BP neural network, which is well applied in geotechnical engineering prediction, was used to predict and evaluate the slope safety of the high-filling channel, and the genetic optimization algorithm (GA) was used to optimize the parameters. Its principle can be referred to in Ref.^41^. A GA-BP neural network model was constructed. The displacements of points A, B, and C were selected for the safety prediction of the high-filling channel slope, and the number of nodes in the hidden layer of the network model was 7, as shown in Fig. 11. The iteration ordinal number of GA was 100 and the population size was 10.

We selected some of the monitoring information in Table 3 and used the Bayesian network and GA-BP neural network model for safety prediction and evaluation. The results are shown in Table 5. According to the time sequence numbers 8, 9, and 10, the evaluation results of the Bayesian network are consistent with the real situation, and the evaluation results of the GA-BP neural network model are too safe and are not in accordance with the real situation. In order to compare the performance of the GA-BP neural network and fusion method based on Bayesian theory, the time sequence number 8 in Table 5 was selected, and the calculated safety factor was compared with the safety factor (1.15) calculated by the actual exploration, determining that the error rate of the Bayesian network was 2.9%, and the error rate of the GA-BP neural network was 42.7%. Comprehensive analysis showed that the fusion method based on Bayesian theory used detection information (as prior information in Bayesian theory) to learn and master things in advance. On this basis, other information was integrated to conduct reasoning and analysis of the evaluation objectives so as to achieve the safety evaluation. The fusion method of the BP neural network used its own learning ability to learn the sample data, and then made a predictive evaluation, the essence of which was to take the multi-source information as the input sample, the target as the output sample, and to establish a high-dimensional nonlinear mapping relationship between the input and the output. In the safety evaluation of the high-filling channel slope, the fusion method of the BP neural network can only learn the computational data of the numerical simulation as training samples, and thus, realize the safety prediction evaluation without integrating the prior information. The fusion method based on Bayesian theory comprehensively considers the computational data based on the cognition of the rock and soil strength parameters on the high-filling channel slope. Compared to neural networks, the problems considered are more comprehensive, and the evaluation results are more reasonable and reliable. The fusion method of the BP neural network appears to be helpless in comprehensively processing prior information and data based on numerical simulations, while Bayesian theory can simply fuse them into a model. In addition, Bayesian theory-based fusion methods are able to characterize the uncertainty to assess the reliability of the evaluation results.

**Table 5:** Comparison of evaluation results between Bayesian network and GA-BP neural network.

Name	Corresponding factor of safety or safety status under different time sequence numbers		
Time sequence number	8	9	10
Bayesian network	1.1834 (mean value)	1.1740 (mean value)	1.1261 (mean value)
GA-BP	1.6410	1.6607	1.6304
Real state	Cracks appear (less safe state: 1.0 < *F*_S_ ≤ 1.2)		

The fusion method based on Bayesian theory requires fewer parameters to be set during the fusion process, and only needs to set the sampling weights and number of samples to generate the initial sample during the MCMC simulation, while the fusion method based on BP neural network needs to set more parameters, including the initial weights, thresholds, and hidden layers, which have a certain impact on the prediction results. Meanwhile, the sample size is also an important factor affecting the prediction accuracy, and a large amount of sample data is difficult to obtain. Neural networks are also prone to falling into local optimal solutions. Although the genetic algorithm can optimize the accuracy of the weights and thresholds of the BP neural network, the genetic algorithm has many parameters to be set, such as the population number, genetic algebra, and so on, the selection of which is often complex and has a great impact on the accuracy of the prediction results. These factors undoubtedly add complexity to the application of neural network methods. In contrast, Bayesian fusion methods require fewer parameters to be set and are more convenient and simpler to apply. Moreover, the increase in the number of samples taken by Bayesian methods improves the accuracy of the evaluation. In summary, the fusion method based on Bayesian theory is simpler and more reliable.

## Conclusions

In this paper, a method of fusing monitoring and detection information based on Bayesian theory is proposed, and the method was applied to the South-to-North Water Diversion Project. The following conclusions can be drawn:

1. The fusion method based on Bayesian theory was applied to the channel of the Central Line Project of South-to-North Water Diversion, and the safety state of the high-filling channel could be captured by fusing monitoring and detection information.
2. The proposed method based on Bayesian theory updates the prior distribution of the cohesion, internal friction angle, and factor of safety after fusing the field monitoring information, and reduces the uncertainty of the interval distribution of the updated variables.
3. In the project, there may be misjudgment if only vertical or horizontal displacement analysis is used to evaluate the safety of the high-filling channel slope, and the evaluation results obtained by combining the two are more consistent with the real state.
4. Compared to the traditional data-driven machine learning method, the evaluation results of the fusion method based on Bayesian theory are more reliable and consistent with the actual state of the on-site engineering.

With the continuous development of science and technology, the monitoring and detection technology of geotechnical engineering is becoming more and more advanced and has higher precision. Monitoring and detection information is also described in various forms, both data and qualitative. The monitoring and detection information fusion method proposed in this paper can realize the information fusion in the data form, but it is difficult to integrate the qualitative information, which is the deficiency of this method, and further research will be done in the future. This method can also be applied to civil engineering, aerospace, and other fields involving data integration.
